# Genome-Wide Association Analyses of Fertility Traits in Beef Heifers

**DOI:** 10.3390/genes12020217

**Published:** 2021-02-02

**Authors:** Morgan R. Stegemiller, Gordon K. Murdoch, Troy N. Rowan, Kimberly M. Davenport, Gabrielle M. Becker, John B. Hall, Brenda M. Murdoch

**Affiliations:** 1 Department of Animal, Veterinary & Food Sciences, University of Idaho, Moscow, ID 83843, USA; mstegemiller@uidaho.edu (M.R.S.); gordon.murdoch@wsu.edu (G.K.M.); kmdavenport@uidaho.edu (K.M.D.); gbecker@uidaho.edu (G.M.B.); 2 Department of Animal Sciences, Washington State University, Pullman, WA 99164, USA; 3 Division of Animal Sciences, University of Missouri, Columbia, MO 65211, USA; tnr343@mail.missouri.edu; 4 Nancy M. Cummings Research, Education, and Extension Center, University of Idaho, Carmen, ID 83462, USA; 5 Center for Reproductive Biology, Washington State University, Pullman, WA 99164, USA

**Keywords:** GWAS, crossbred beef heifers, imputed, antral follicle count, reproductive tract score

## Abstract

The ability of livestock to reproduce efficiently is critical to the sustainability of animal agriculture. Antral follicle count (AFC) and reproductive tract scores (RTS) can be used to estimate fertility in beef heifers, but the genetic mechanisms influencing variation in these measures are not well understood. Two genome-wide association studies (GWAS) were conducted to identify the significant loci associated with these traits. In total, 293 crossbred beef heifers were genotyped on the Bovine GGP 50K chip and genotypes were imputed to 836,121 markers. A GWAS was performed with the AFC phenotype for 217 heifers with a multi-locus mixed model, conducted using the year, age at time of sampling and principal component analysis groupings as the covariates. The RTS GWAS was performed with 289 heifers using an additive correlation/trend test comparing prepubertal to pubertal heifers. The loci on chromosomes 2, 3 and 23 were significant in the AFC GWAS and the loci on chromosomes 2, 8, 10 and 11 were significant in the RTS GWAS. The significant region on chromosome 2 was similar between both analyses. These regions contained genes associated with cell proliferation, transcription, apoptosis and development. This study proposes candidate genes for beef cattle fertility, although future research is needed to elucidate the precise mechanisms.

## 1. Introduction

Cattle producers benefit from animals that reproduce reliably and efficiently, making fertility a critical trait in the cattle industry. Improving reproductive efficiency can be accomplished by selecting replacement heifers with higher fertility and a longer reproductive life span. There are several measures used to estimate fertility, including Anti-Müllerian hormone concentration, days open and calving performance: this study focuses on the antral follicle count (AFC) and reproductive tract scores (RTS).

Antral follicle count is highly repeatable within individuals although it can vary within a population [1]. The fertility measurement of AFC has been utilized in cattle and requires only a single, non-invasive ultrasound examination [2]. The heritability of AFC in cattle is 0.25 and is positively correlated with other indirect measures of fertility, such as endometrial thickness, super ovulatory response and herd longevity [1,2,3,4,5,6]. In addition, cattle with a higher AFC tend to have ovaries of significantly greater length, size and weight [7]. Ovaries with a greater number of antral follicles indicate a larger ovarian reserve (OR) [8]. A smaller OR has been associated with reduced ovarian function [9]. Although the proportion of healthy to total number of follicles is the same between animals with either high or low AFC, the total number of healthy follicles is greater in those with high AFC (>25) [7]. Furthermore, *Bos taurus*, *Bos indicus* and *Bos indicus-taurus* animals that have higher AFC also produce more oocytes during superovulation [2,4,5]. Previous studies in *Bos taurus* crossbred beef heifers and Holstein-Friesian dairy cows have shown that animals with a high AFC are more likely to be pregnant at the end of breeding season [8,10].

In addition to AFC, producers use RTS as a semi-objective measurement of pubertal status and to determine the age of puberty that has a heritability of 0.43 [11]. Briefly, scoring is based on palpation of follicular development, corpus luteum presence and reproductive tract tone. A scale from one to five is used, in which a score of one indicates the animal is immature or in anestrous while five shows the animal is mature and cycling, as described previously [12]. Unsurprisingly, heifers with a higher RTS are more likely to conceive and to conceive earlier in the breeding season than heifers with a lower RTS [13].

Understanding the biological mechanisms contributing to increased AFC and earlier reproductive tract development will allow producers to select for more reproductively efficient animals as replacements in their herd. This study used genome-wide association studies (GWAS) to investigate if AFC and RTS exhibit significant genetic associations with genetic variation and has identified the potential biological pathways involved. Previous GWAS in cattle, such as Neupane et al. and Cole et al., have identified the genes associated with fertility and reproductive traits in cattle; however, neither examined AFC or RTS [14,15]. Determining the mechanisms contributing to follicular and reproductive tract development will provide a basis for future fertility studies.

## 2. Materials and Methods

### 2.1. Animals and Phenotype Collection of Antral Follicle Count and Reproductive Tract Scores

This study examined a total of 293 crossbred heifers over a two-year period, 139 from year one and 154 from year two. The heifers were sired by Angus, Hereford, Simmental, Simmental-Angus (SimAngus) or Shorthorn bulls. These animals were raised at the University of Idaho Nancy M. Cummings Research, Education and Extension Center in Carmen, Idaho. The heifers ranged from 10.5 to 13.5 months of age and had a body condition score between five and seven at the time of evaluating the antral follicles and reproductive tracts. AFC data were collected on heifers (*n* = 220) by performing ovarian ultrasound imaging with an Ibex EVO portable ultrasound with a 7.5 MHz linear probe [7,10]. The recorded ultrasounds were examined to identify follicles ≥3 mm, which were counted for the total AFC [16]. Reproductive tracts were scored in heifers (*n* = 293) using palpation and confirmed with ultrasound [12,16]. Data from the first year of heifers have been previously published by Reynolds et. al in 2018 [16].

### 2.2. Genotyping

Blood was collected from 293 heifers at NMCREEC and shipped to the University of Idaho where DNA was isolated using the phenol chloroform method as previously described [17]. DNA for each animal was genotyped with the Bovine GGP 50K chip that consisted of 47,843 Single Nucleotide Polymorphism (SNP) markers (Neogen, Lincoln, NE, USA). In total, 293 samples were genotyped; however, four samples were removed as they had a call rate <0.9. Therefore, 289 samples were analyzed; however, only a subset of these samples (*n* = 217) had AFC data. Further, non-autosomal markers and those with a call rate <0.9 were removed. The remaining 45,436 variants were phased using Eagle (v2.4.1) [18], and then imputed up to 836,121 SNP markers with Minimac3 [19] using the methods and reference panels described in Rowan et al. [20]. Briefly, the imputation reference panel contained 9629 animals genotyped on the Illumina HD array (777K SNPs), 28,183 animals genotyped on the GGP-F250 array (~227K SNPs) and 2718 animals genotyped on both high-density assays. The multi-breed imputation reference contained between 354 (Shorthorn) and 16,703 (Angus) high-density individuals from the component breeds represented in the genotyped dataset. Rowan et al. (2019) observed high individual imputation accuracies (r^2^ > 0.99) for crossbred animals using this same multi-breed reference panel [20]. After the imputation, SNPs with a minor allele frequency (MAF) <0.01 were discarded and the remaining 712,666 SNPs were used in the subsequent analyses.

### 2.3. Genotypic Analyses

A principal component analysis (PCA) was performed to examine the genetic relatedness of heifers. A Fisher’s Exact Test was run in R version 3.6.2 and used to determine if the PCA groups had a difference in proportion of heifers classified in the high, medium and low AFC groups [21]. An association analysis was performed for 217 heifers with AFC data. A kinship matrix was first created to correct for any population structure that may exist in the sample set. Year of collection, age at time of AFC (to the nearest half month) and PCA groupings were used as covariates. A multi-locus, mixed-model additive association test was performed [22]. A separate association test with 289 heifers and RTS phenotype was also performed. For this GWAS, RTS of 1 and 2 (prepubertal heifers) were classified as cases, and RTS of 3–5 were classified as controls. Subsequent to a genetic relationship matrix correction, an additive correlation/trend association test was performed. The SNP & Variation Suite^TM^ software, version 8.7.2, was used for the PCA and GWAS analyses (Golden Helix, Inc., Bozeman, MT, USA; www.goldenhelix.com). Genome-wide significance threshold of *p* < 1.00 × 10^−5^ was set based on previous species-specific research [23,24,25]. 

## 3. Results

### 3.1. Principal Component Analysis

**Figure 1:**
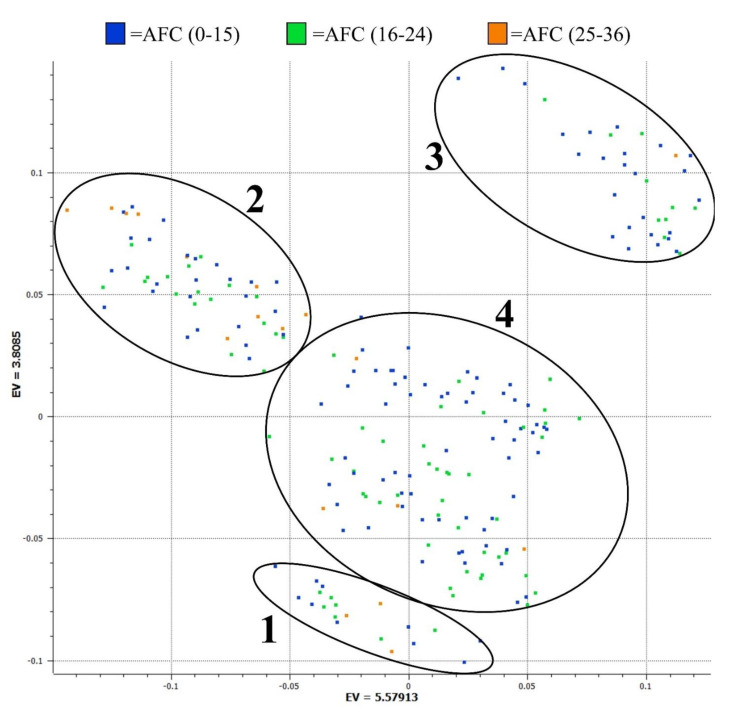
Principal component analysis plot to group animals by genotype by plotting the two largest eigenvectors (EV). Group 1 are heifers of a single SimAngus sire; Group 2 are all Hereford-sired heifers; Group 3 are heifers of a single Angus sire; and Group 4 are the heifers of the remining Angus, Simmental, SimAngus and Shorthorn sires. The individual heifers are color coded based on the number of the antral follicle count (AFC) observed, with blue exhibiting lower (0–15), green medium (16–24) and orange the highest number of (25–36) the AFC observed.

In order to examine the genetic relationship of the heifers used in the AFC analysis, we performed a principal component analysis (PCA) and plotted the first two eigenvalues. The PCA plot depicts that the heifers separate into four distinct groups (Figure 1). Group 1 consists of heifers that were sired by a single SimAngus sire and Group 2 are heifers sired by six Hereford bulls. Group 3 consists of heifers sired by a single Angus bull and Group 4, the largest group, consists of heifers out of 18 Angus, SimAngus, Simmental and Shorthorn sires. Groups 1 and 3 are comprised of heifers that belong in the year two cohort, and their sires were not used previously for year 1. Groups 2 and 4 consist of heifers sired by animals that were used for both years. The individual heifers in this plot are colored based on AFC categories of low (≤15), medium (16–24) and high (≥25) [16]. Interestingly, heifers in the high AFC category are not evenly distributed between the PCA groups. Over half (55%) of all high AFC heifers are grouped in PCA Group 2, and although PCA Group 4 contains 50% of the population, only 22% of the Group 4 heifers had a high AFC. Using a Fisher’s Exact Test, there was a significant (*p* = 0.004) difference in the proportion of heifers classified as high AFC vs. those classified as medium and low between the PCA groups.

### 3.2. GWAS with Antral Follicle Count 

**Figure 2:**
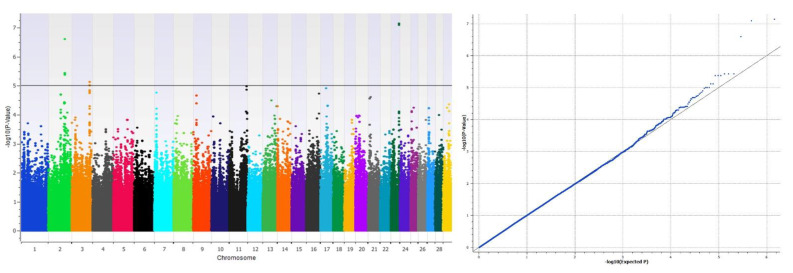
Manhattan plot of the GWAS for variation of the antral follicle count (AFC) in heifers in the left panel and the corresponding qq plot in the right panel. The solid line denotes genome-wide significance at −log(*p*-value) = 5.

A GWAS was performed to test for genetic associations in 217 heifers with different AFCs. Chromosomes 2, 3 and 23 exhibited loci that are significantly associated with differences in AFC (Table 1). In total there were 14 significant SNPs, with seven on chromosome 2, five on chromosome 3 and two SNPs on chromosome 23. The results from the GWAS are displayed in a Manhattan plot in Figure 2. 

The percentage of variance explained (PVE) identified in Table 1 was calculated in SVS to show how much variation can be explained by the effects of the marker [22]. The two significant SNPs on chromosome 23 explained most of the variation at 12.9% with the seven SNPs on chromosome 2 explaining 11.9% of the variation. Further, the five SNPs on chromosome 3 explained 9.1%. In relation to the reference genome ARS-UCD 1.2, the homozygous reference markers on chromosomes 2 and 23 are associated with increased AFC while the reference markers on chromosome 3 are associated with decreased AFC [26].

**Figure 3:**
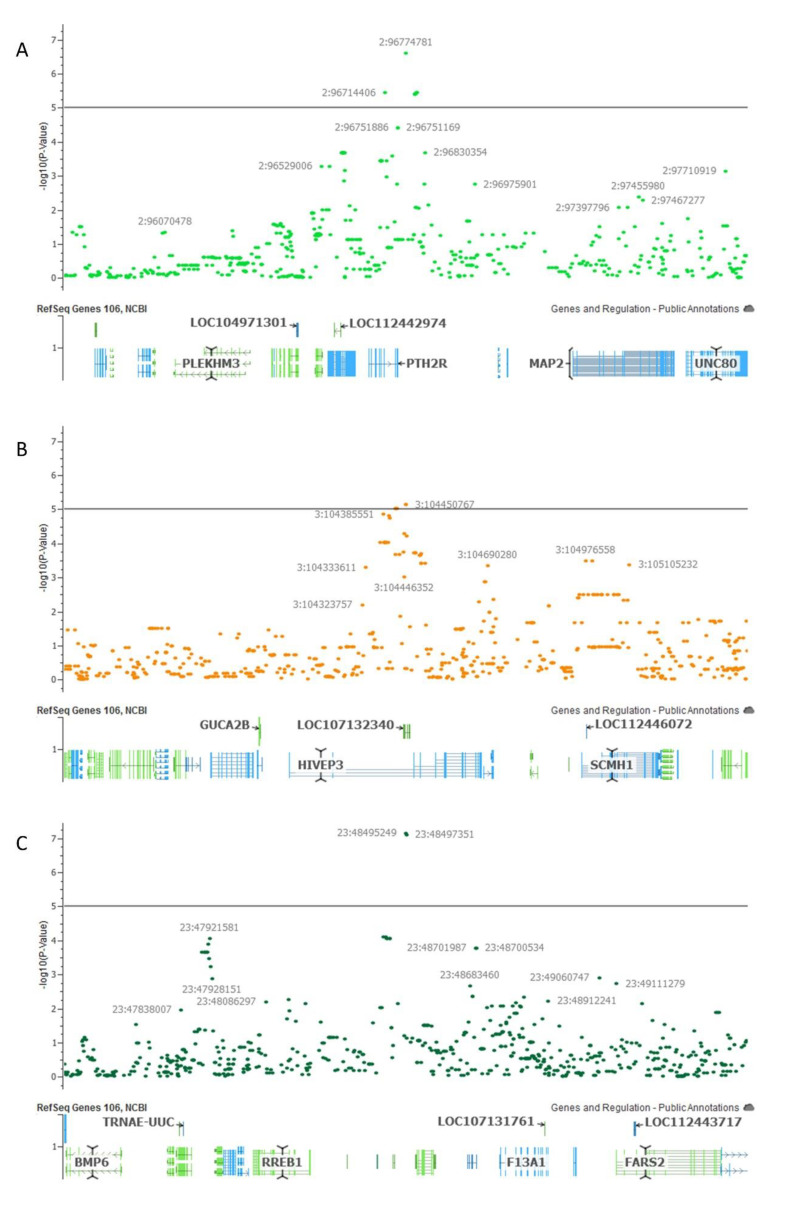
Regions within 1 Mbp of the most significant SNP for the antral follicle count GWAS results for (**A**) chromosome 2; (**B**) chromosome 3; (**C**) chromosome 23.

Subsequently, annotated genes within 1 Mb upstream and downstream of significant SNPs were identified. An enlarged view of the genomic regions containing significant SNPs as well as all markers within 1 Mb upstream and downstream are illustrated in Figure 3. Within this region, there are a total of 12 genes located on chromosome 2, 22 genes on chromosomes 3 and 13 genes on chromosome 23 (Supplemental Table S1). 

The genes in the regions of interest identified as candidate genes are noted in Table 1. The genes were categorized as candidate genes because they have been identified in pathways that have known effects or the potential to affect follicle development. The most significant region on chromosme 23 had two candidate genes identified. On chromosome 2, a total of six candidate genes were identified while on chromosome 3 one gene was identified. Of these nine identified genes, six are currently known to contribute to or affect the process of three well-known biological pathways linked to reproduction. These pathways are the PI3K/AKT, WNT signaling and MAP kinase (MAPK) pathways, all of which have been identified as affecting folliculogenesis and demonstrates prior knowledge of candidate gene pathways. 

### 3.3. GWAS with Reproductive Tract Scores

**Figure 4:**
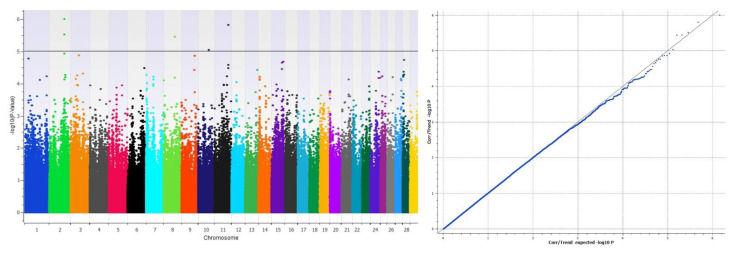
Manhattan plot of the GWAS for the variation in reproductive tract scores (RTS) in heifers in the left panel and the corresponding qq plots in the panel on the right. The solid line is at significance level –log(*p*-value) = 5.

A separate GWAS was performed using 289 heifers to investigate genetic associations with the RTS phenotypes (Figure 4). The focus of this analysis was to examine the pre-pubertal animals in comparison to the pubertal heifers. There are four significant loci above the genome-wide significance threshold of *p* < 1.00 × 10^−5^. Chromosomes 2 and 8 had two significant SNPs each and chromosomes 10 and 11 had one significant SNP each (Table 2). There was the same area of significance between the two fertility traits on chromosome 2 at 96.8 Mb.

**Figure 5:**
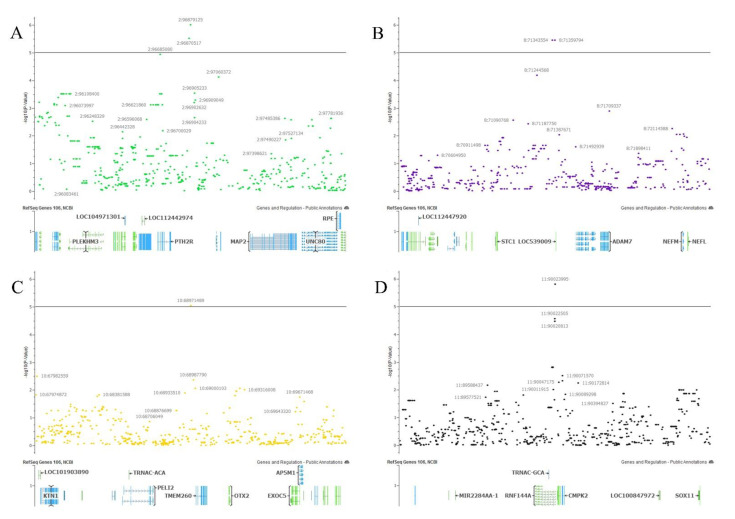
Regions within 1 Mbp of the most significant SNP for the reproductive tract scores (RTS) GWAS results for (**A**) chromosome 2; (**B**) chromosome 8; (**C**) chromosome 10; (**D**) chromosome 11.

From these four locations, regions of significance were defined as 1 Mb upstream and downstream of each significant SNP. These regions were investigated using the ARS-UCD 1.2 reference genome (Figure 5) [26]. The significant SNPs identified were in gene-rich regions. In the significant regions there are a total of 14 genes on chromosome 2, 11 genes on chromosome 8, nine genes on chromosome 10 and four genes on chromosome 11 (Supplemental Table S2). 

Of the genes listed in the regions of interest, 15 genes were identified as candidate genes (Table 2). The genes were identified as candidate genes because they have been identified in pathways that have known effects or the potential to affect the development of the reproductive tract. From the 15 candidate genes, six are located in proximity with the most significant loci on chromosome 2. These six genes on chromosome 2 are located in the proximity of significant SNPs identified in both the AFC and RTS GWASs. The other significant regions on chromosome 8, 10 and 11 have three candidate genes each for a total of nine candidate genes. Two of these candidate genes are involved with pathways previously mentioned, the WNT signaling and the MAPK pathway.

## 4. Discussion

Fertility is a highly variable trait but is of major importance in the cattle industry. Cattle fertility can be measured by AFC and RTS, although little is understood of the genetic variation associated with these measurements. In order to examine the genetic associations with these traits we conducted two GWAS using crossbred heifers.

### 4.1. GWAS Related to Antral Follicle Count

**Figure 6:**
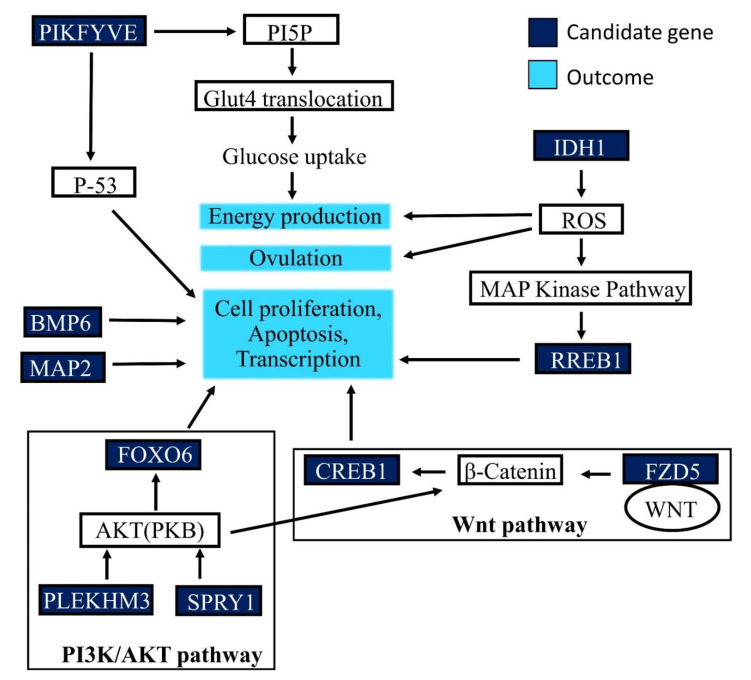
Proposed pathway(s) for the candidate genes in regions of the significant SNPs associated with antral follicle count (AFC). Arrows indicate an effect not increasing or decreasing.

The genes located within the significant regions of the AFC GWAS that have previously been described in important biological pathways are reported in Figure 6. One notable pathway is the PI3K/AKT pathway, as it is known to be involved in the activation of AKT in granulosa cells in rats and we can predict a synonymous role in the granulosa cells of cattle [27]. Two candidate genes identified in this study, pleckstrin homology domain containing M3 (*PLEKHM3*) and forkhead box O6 (*FOXO6*), are closely related to the PI3K/AKT pathway. *PLEKHM3* codes for a scaffold protein for AKT that localizes AKT to the plasma membrane, an essential step in AKT activation, whereas FOXO6 is a transcription factor that is regulated by AKT [28,29]. Little is known about FOXO6 in tissues other than the brain; however, other genes in this family are involved in cell metabolism and death of oocytes [30,31]. 

The candidate gene phosphoinositide kinase, FYVE-type zinc finger containing protein (*PIKFYVE*) directly affects the level of the cellular phosphoinositides, PI(3,5)P2 and PI5P [32]. When the PI5P levels increase, this triggers the translocation of glucose transporters to the cell membrane, which leads to an increase in intracellular glucose (energy) levels [33]. PI5P further regulates the p-53 dependent apoptotic pathway, and it is plausible that preventing apoptosis supports folliculogenesis and maturation [33]. *PIKFYVE* has been identified as a putative target of microRNA that is differentially expressed in dominant theca cells [34]. It is conceivable that the association of this pathway with reproductive traits relates to the influence on cellular energy levels as well as regulation of apoptosis.

The WNT signaling pathway contributes to oocyte development [35]. The canonical WNT pathway uses β-catenin to affect gene transcription [30]. Previous studies show that WNT proteins are required for ovarian follicle development via regulating FSH and LH signaling [36]. WNT2 induces an accumulation of β-catenin that through the canonical pathway causes an increase in estradiol production [35]. Alternatively, for normal female fertility, WNT5a suppresses the canonical pathway [36]. The critical role of Wnt5a in folliculogenesis is supported by the report that Wnt5a-null mice experience an increased rate of follicular atresia [36]. Two candidate genes are involved in this pathway, frizzled receptor 5 (*FZD5*) and cAMP responsive element binding protein (*CREB1*). FZD5 is a receptor for several WNT ligands, including WNT5a and WNT2. β-catenin is also affected by the PI3K/AKT pathway mentioned previously. β-catenin can be phosphorylated by AKT, which increases its transcriptional activity [37]. CREB1 is associated with the canonical pathway and is shown to have decreased levels in this process when the canonical pathway is suppressed [36]. *CREB1* is also a putative target of microRNA that is differentially expressed in the dominant granulosa cells [34].

The candidate gene isocitrate dehydrogenase 1 (*IDH1*) codes for a protein involved in the TCA cycle and regulates oxidoreductase activity as well as metabolism. A study in rats engineered to mimic PCOS saw a reduction in *IDH1* levels, indicating that *IDH1* is involved in follicular growth and ovulation [38]. Due to its involvement in the TCA cycle, *IDH1* may affect the energy and reactive oxygen species (ROS) levels [39]. ROS are necessary to induce ovulation [39]. In addition, ROS are mediators of the MAPK pathway, which has been shown to affect follicle maturation [39,40]. Another candidate gene ras responsive element binding protein (*RREB1*) is a downstream effector of the MAPK [41]. Overall, changes in ROS levels can affect the outcomes of the MAPK pathway, thereby influencing cell proliferation and the likelihood of ovulation. 

The last two candidate genes are bone morphogenic protein-6 (*BMP6*) and microtubule-associated protein 2 (*MAP2*). Several bone morphological proteins and their receptors are involved of cell proliferation, apoptosis and cell differentiation [42]. In the ovary, *BMP6* works to regulate FSH through decreasing the cAMP levels [43]. The last gene, *MAP2*, codes for a protein that helps to stabilize microtubules during growth, a step which is important for proper separation during mitosis [44]. This protein is also shown to be upregulated as follicles mature normally from primordial to secondary follicles in mice [45].

A study by Fortes et. al examined genes associated with puberty in tropical breeds of cattle [46]. The authors of this study identified two genes, HIVEP zinc finger 3 (*HIVEP3*) and *RREB1*, which are also found in our regions of significance for our AFC GWAS on chromosome 3 and chromosome 23, respectively. The previous association of these regions with onset of puberty supports our findings with fertility traits in beef cattle.

Identifying the underlying genetic variation that contributes to the biological mechanisms of AFC can help improve knowledge of the aspects of germ cell development that are currently not understood. Previous studies have enumerated the benefits of animals that present a high AFC. However, more recent studies in various dairy and *Bos indicus* animals have shown contrasting results. In a study with Holsteins, heifers in the high AFC group had more days open and less percent pregnant at the end of lactation than either the low or medium groups [47]. Similarly, a study in Nelore cattle reports the highest conception rates in animals with the lowest AFC, and the lowest conception rates in animals with the highest AFC [48].

One possible explanation is that some animals with high AFC have polycystic ovary syndrome (PCOS), which negatively impacts fertility and skews the relationship between follicle number and fertility [47]. Females with PCOS may have a higher AFC, but chronic anovulation results in subfertility [49,50]. In addition, the question remains as to whether increased AFC indicates a longer reproductive lifespan or alternatively that the OR is depleted more quickly [51]. This study has identified nine candidate genes that are associated with differences in AFC. These genes also warrant further investigation with respect to their influence and associations with PCOS.

### 4.2. GWAS Related to Reproductive Tract Scores (RTS)

This study identifies regions on four chromosomes that are significantly associated with the RTS. The region on chromosome 2 is the same region also associated with AFC. A component of RTS determination is ovarian structure and either the presence or absence of ovulating follicles. Thus, it is reasonable to have the same region be significant as both GWAS include follicle development. Two candidate genes identified in the RTS GWAS influence the pathways previously discussed the context of AFC. These include Pellino2 (*PELI2*), which is associated with the MAPK pathway, and sex-determining region Y-box transcription factor 11 (*SOX11*), which is associated with the WNT signaling pathway [52]. PELI2 can activate the MAPK pathway, which, as previously discussed, can affect the maturation of ovarian follicles [40,53]. *SOX11* is expressed in ovarian cells and can dampen the WNT signaling pathway [52,54]. As mentioned previously, WNT proteins are crucial to regulate FSH and LH signaling for follicle development [36]. 

Multiple candidate genes also affect follicle maturation in pathways not previously discussed. Although first identified in maintenance of mineral levels, Stanniocalcin (*STC1*) has been shown to be highly detectable in the ovaries of mature mice [55]. It has been reported that overexpression of *STC1* in mice is significantly associated with a reduction in litter size [56]. Although this mechanism remains unclear, it is evident that *STC1* affects reproduction in mammals. RNF144A is a transmembrane ligase that interacts with epidermal growth factor receptor (EGFR) and thereby affects EGFR-stimulated cell proliferation [57]. Previous studies in mice demonstrate that EGFR is required for cumulous-oocyte complex maturation [58]. Through this interaction, changes in RNF144A can affect the maturation and development of follicles. 

Several of these candidate genes affect mammalian reproductive ability. Lysyl oxidase-like protein-2 (*LOXL2*) functions in extracellular matrix production by collagen IV assembly [59]. *LOXL2* has been identified in human female reproductive tracts and in cells with advanced aging so its presence in mammalian reproductive tracts is important in our study when comparing heifers that mature less quickly than their cohorts [60,61]. Orthodenticle homeobox 2 (*OTX2*) affects the GnRH, LHB and FSHB levels in pituitary tissues of mice. In addition, mice without Otx2 had delayed vaginal opening and fewer litters than mice with a functioning Otx2 [62]. It is known that mice without Otx2 have smaller litters, abnormal estrous cycles and lack of corpus lutea [62]. Any change in OTX2 could affect not only follicle development and ovulation but also reproductive physiology.

Two final genes of note in our significant region: transmembrane protein 260 (*TMEM260*) and cytodine/uridine monophosphate kinase 2 (*CMPK2*). *TMEM260* is a putative transmembrane protein with an unknown function. According to NCBI, the expression of *TMEM260* is high in ovaries, implying a function in mammal reproduction [63]. *CMPK2* is a mitochondrial protein that phosphorylates dCMP and dUMP during mtDNA synthesis; however, it has not been observed in all tissues [64,65]. *CMPK2*, as it functions in the mitochondria, may be involved in the regulation of essential nucleotide during mitochondrial biogenesis and influence energy production and growth [64].

## 5. Conclusions

The GWAS identified three regions significantly associated with the AFC and four associated with the RTS in crossbred heifers. One region on chromosome 2 is significantly associated with both fertility phenotypes and contains six candidate genes. In addition to these six genes, three candidate genes relating to AFC are associated with pathways that contribute to follicle growth through energy production, cell proliferation, transcription or apoptosis. In addition to the six candidate genes on chromosome 2, nine more candidate genes are in proximity to the regions associated with RTS and could affect the reproductive development of heifers. This study reports an association between these regions and AFC or RTS; however, additional work with larger sample sizes is needed to establish causal variants and the mechanisms of the effects they have on follicle development and fertility in cattle.

## Figures and Tables

**Table 1:** Chromosome, rs number, position, *p*-value, −log(*p*-value), percentage of variance explained, and the candidate genes for significant SNPs associated with the AFC.

Chromosome	rs Number	Position (bp)	*p*-value	−log(*p*-value)	Percentage of Variance Explained (%)	Candidate Genes
23	42333762	48495249	7.20 × 10^−8^	7.14	12.9	*BMP6*, *RREB1*
	42333752	48497351	8.03 × 10^−8^	7.10	12.8	
2	110145174	96774781	2.53 × 10^−7^	6.60	11.9	*CREB1*, *FZD5*, *PLEKHM3*, *IDH1*, *PIKFYVE, MAP2*
	109967601	96714406	3.67 × 10^−6^	5.43	9.7	
	109066756	96804827	3.68 × 10^−6^	5.43	9.7	
	109574474	96807982	3.68 × 10^−6^	5.43	9.7	
	110145277	96801078	4.16 × 10^−6^	5.38	9.6	
	109609461	96801273	4.16 × 10^−6^	5.38	9.6	
	135894326	96802055	4.16 × 10^−6^	5.38	9.6	
3	133573457	104450767	7.58 × 10^−6^	5.12	9.1	*FOXO6*
	43366810	104451813	7.58 × 10^−6^	5.12	9.1	
	43367756	104420695	9.79 × 10^−6^	5.01	8.9	
	110027403	104422767	9.79 × 10^−6^	5.01	8.9	
	43367746	104423898	9.79 × 10^−6^	5.01	8.9	

**Table 2:** Chromosome, rs number, position, *p*-value, –log(*p*-value) and candidate genes for significant SNPs associated with the RTS.

Chromosome	rs Number	Position (bp)	*p*-value	−log(*p*-value)	Candidate Genes
2	110658876	96879125	9.99 × 10^−7^	6.00	*CREB1*, *FZD5*, *PLEKHM3*, *IDH1*, *PIKFYVE*, *MAP2*
	136255286	96870517	3.05 × 10^−6^	5.52	
8	110172413	71343554	3.56 × 10^−6^	5.45	*LOXL2*, *STC1*
	137380598	71359794	3.56 × 10^−6^	5.45	
10	134739799	68971489	9.11 × 10^−6^	5.04	*PELI2*, *TMEM260*, *OTX2*
11	111004666	90023995	1.55 × 10^−6^	5.81	*RNF144A*, *CMPK2*, *SOX11*

## Data Availability

Data will be available upon request.

## Supplementary Materials

The following are available online at https://www.mdpi.com/2073-4425/12/2/217/s1, Table S1: Genes in Significant Region for AFC, Table S2: Genes in Significant Region for RTS
