# Supplementary material for: Genome-Wide Association Analyses of Fertility Traits in Beef Heifers
Source: Genes (Basel). 2021 Feb 2;12(2):217. doi: 10.3390/genes12020217 (PMC7913221; doi:10.3390/genes12020217)
Supplement: Supplementary file 1 [file genes-12-00217-s001.gz › Supplementary.docx]

Table S1: Location of significant SNPs and the genes located within 1 Mbp of the most significant SNP in the chromosomal region for AFC

| Chromosome | Significant SNP Locations | Gene | Gene Location |
| --- | --- | --- | --- |
| 2 | 96,774,781 96,714,406^a^ 96,804,827 96,807,982 96,801,078 96,801,273 96,802,055 | *CREB1* | 95864818-95897170 |
|  |  | *METTL21A* | 95908907-95918927 |
|  |  | *CCNYL1* | 95992629-96029603 |
|  |  | *FZD5* | 96034762-96042102 |
|  |  | *PLEKHM3* | 96095228-96318519 |
|  |  | *CRYGA-D* | 96395739-96435050 |
|  |  | *C2H2oef80* | 96437056-96460429 |
|  |  | *IDH1* | 96510381-96531400 |
|  |  | *PIKFYVE* | 96546574-96628760 |
|  |  | *PTH2R* | 96667750-96751783 |
|  |  | *MAP2* | 97263427-97562142 |
|  |  | *UNC80* | 97594858-97825043 |
| 3^b^ | 104,450,767 104,451,813 104,420,695 104,422,767 104,423,898 | *YBX1* | 103520065-103539625 |
|  |  | *PPIH* | 103540363-103565504 |
|  |  | *LOC 112445912* | 103568648-103580856 |
|  |  | *CCDC30* | 103584645-103713873 |
|  |  | *ZMYND12* | 103720824-103755638 |
|  |  | *PPCS* | 103716209-103720494 |
|  |  | *RIMKLA* | 103772438-103807695 |
|  |  | *LOC101907688* | 103807843-103851717 |
|  |  | *F0XJ3* | 103880144-104005470 |
|  |  | *GUCA2A* | 104015493-104031213 |
|  |  | *GUCA2B* | 104021061-104024316 |
|  |  | *HIVEP3* | 104109087-104674365 |
|  |  | *LOC107132340* | 104442728-104464286 |
|  |  | *EDN2* | 104679329-104706954 |
|  |  | *FOXO6* | 104815304-104837023 |
|  |  | *SCMH1* | 104964994-105189644 |
|  |  | *SLFNL1* | 105189750-105198784 |
|  |  | *CTPS1* | 105201090-105232559 |
|  |  | *CITED4* | 105348797-105350138 |
|  |  | *KCNQ4* | 105374630-105433410 |
|  |  | *MIR30C,E* | 105457746-105460971 |
|  |  | *NFYC* | 105445059-105510431 |
| 23 | 48,495,249 48,497,351 | *BMP6* | 47502057-47662629 |
|  |  | *SNRNP48* | 47797829-47816534 |
|  |  | *DSP* | 47824109-47868043 |
|  |  | *CAGE1* | 47960663-47997767 |
|  |  | *RIOK1* | 47936864-47960629 |
|  |  | *SSR1* | 48004708-48034860 |
|  |  | *RREB1* | 48048382-48215277 |
|  |  | *LY86* | 48528635-48576190 |
|  |  | *LOC112443817* | 48676267-48702082 |
|  |  | *F13A1* | 48770276-48913854 |
|  |  | *NRN1* | 48986030-48994877 |
|  |  | *FARS2* | 49111514-49417402 |
|  |  | *LYRM4* | 49417555-49509666 |
| a. SNP on chromosome 2 at location 96714406 is located within PTH2R gene | | | |
| b. All five SNPS on Chromosome 3 located within HIVEP3 gene | | |  |

Supplemental table 2: Location of significant SNPs and the genes located within 1 Mbp of the most significant SNP in the chromosomal region for RTS

| Chromosome | Significant SNP Locations | Gene | Gene Location |
| --- | --- | --- | --- |
| 2 | 96,870,517 96,879,125 | *CREB1* | 95864818-95897170 |
|  |  | *METTL21A* | 95908907-95918927 |
|  |  | *CCNYL1* | 95992629-96029603 |
|  |  | *FZD5* | 96034762-96042102 |
|  |  | *PLEKHM3* | 96095228-96318519 |
|  |  | *CRYGA-D* | 96395739-96435050 |
|  |  | *C2H2oef80* | 96437056-96460429 |
|  |  | *IDH1* | 96510381-96531400 |
|  |  | *PIKFYVE* | 96546574-96628760 |
|  |  | *PTH2R* | 96667750-96751783 |
|  |  | *MAP2* | 97263427-97562142 |
|  |  | *UNC80* | 97594858-97825043 |
|  |  | *RPE* | 97826468-97847708 |
|  |  | *KANSL1L* | 97846946-97972643 |
| 8 | 71,343,554 71,359,794 | *CHMP7* | 70374034-70387496 |
|  |  | *R3HCC1* | 70404873-70413064 |
|  |  | *LOXL2* | 70414275-70527420 |
|  |  | *ENTPD4* | 70548411-70586238 |
|  |  | *SLC25A37* | 70670484-70711535 |
|  |  | *NKX2.6* | 70823594-70828041 |
|  |  | *NKX3.1* | 70788982-70791605 |
|  |  | *STC1* | 70977493-70990673 |
|  |  | *ADAMDEC1* | 71496993-71622274 |
|  |  | *ADAM7* | 71653464-71706037 |
|  |  | *NEFM* | 72181208-72186703 |
|  |  | *NEFL* | 72213269-72217537 |
| 10 | 68,971,489 | *KTN1* | 68006271-68119073 |
|  |  | *PELI2* | 68531911-68731213 |
|  |  | *TMEM260* | 69005149-69075631 |
|  |  | *OTX2* | 69215035-69224752 |
|  |  | *EXOC5* | 69613361-69671092 |
|  |  | *AP5M1* | 69671083-69696378 |
|  |  | *NAA30* | 69768396-69789540 |
|  |  | *CCDC198* | 69823216-69853335 |
|  |  | *SLC35F4* | 69899835-69931916 |
| 11 | 90,023,995^a^ | *RNF144A* | 89897594-90026495 |
|  |  | *RSAD2* | 90038987-90056291 |
|  |  | *CMPK2* | 90068004-90085403 |
|  |  | *LOC100847972* | 90692236-90701372 |
|  |  | *SOX11* | 90946223-90955282 |
| a. The SNP marker on chromosome 11 at 90023995 is located within the RNF144A gene. | | | |


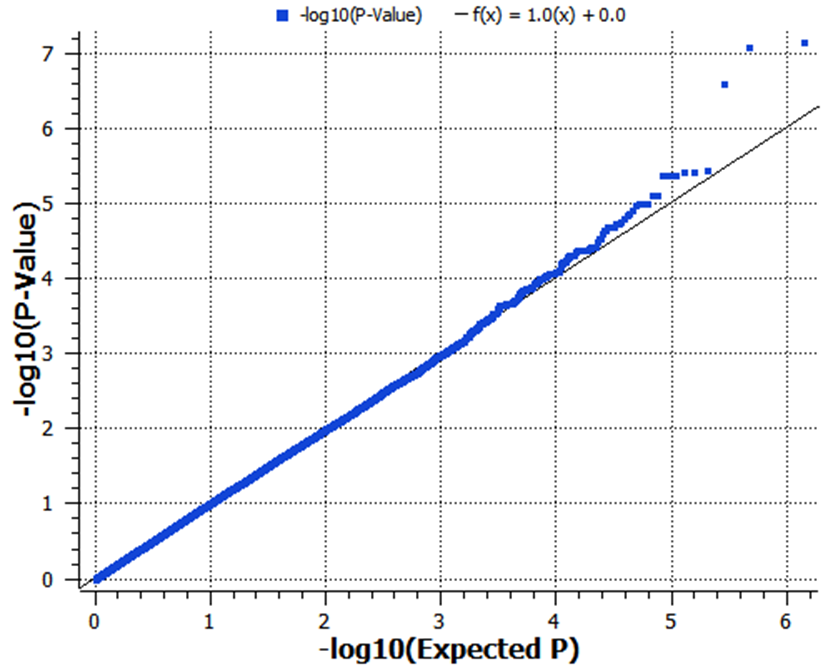


Figure S1: QQ plot that corresponds to the Multi-locus mixed model Manhattan plot for AFC


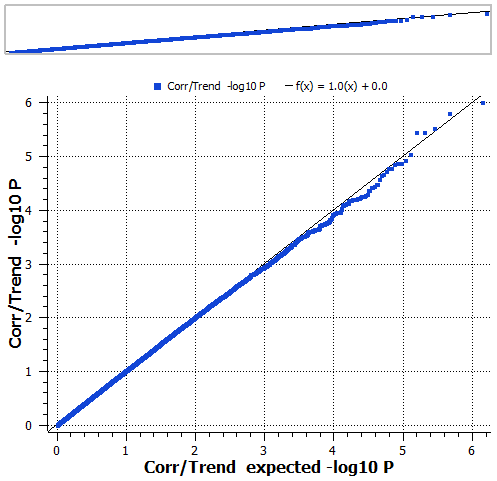


Figure S2: QQ plot that corresponds to the Correlation/Trend Manhattan plot for RTS
